# Antibody‐mediated inhibition of tissue‐type plasminogen activator binding to the low‐density lipoprotein receptor‐related protein 1 as a potential beneficial modulator for stroke therapy

**DOI:** 10.1002/jcb.30431

**Published:** 2023-06-08

**Authors:** Philip H. Klecker, Laura Fritzen, Alexander D. Mazura, Sascha Weggen, Claus U. Pietrzik

**Affiliations:** ^1^ Institute for Pathobiochemistry University Medical Center of the Johannes Gutenberg‐University Mainz Germany; ^2^ Institute of Neuropathology Heinrich‐Heine‐University Düsseldorf Germany

**Keywords:** blood‐brain barrier (BBB) integrity, Claudin‐5, low density lipoprotein receptor‐related protein 1 (LRP1), matrix metalloproteinase 9 (MMP9), microglia, Tissue‐type plasminogen activator (tPa)

## Abstract

The acute ischemic stroke therapy of choice is the application of Alteplase, a drug containing the enzyme tissue‐type plasminogen activator (tPa) which rapidly destabilizes blood clots. A central hallmark of stroke pathology is blood‐brain barrier (BBB) breakdown associated with tight junction (TJ) protein degradation, which seems to be significantly more severe under therapeutic conditions. The exact mechanisms how tPa facilitates BBB breakdown are not entirely understood. There is evidence that an interaction with the lipoprotein receptor‐related protein 1 (LRP1), allowing tPa transport across the BBB into the central nervous system, is necessary for this therapeutic side effect. Whether tPa‐mediated disruption of BBB integrity is initiated directly on microvascular endothelial cells or other brain cell types is still elusive. In this study we could not observe any changes of barrier properties in microvascular endothelial cells after tPa incubation. However, we present evidence that tPa causes changes in microglial activation and BBB breakdown after LRP1‐mediated transport across the BBB. Using a monoclonal antibody targeting the tPa binding sites of LRP1 decreased tPa transport across an endothelial barrier. Our results indicate that limiting tPa transport from the vascular system into the brain by coapplication of a LRP1‐blocking monoclonal antibody might be a novel approach to minimize tPa‐related BBB damage during acute stroke therapy.

**Abbreviations**
BBB: blood‐brain‐barrier
BMECs: brain mouse endothelial cells
Iba‐1: ionized calcium‐binding adapter molecule 1
LRP1: low‐density lipoprotein receptor‐related protein 1
MMP: matrix metalloproteinase
RAP: receptor‐associated protein
TEER: transendothelial electrical resistance
TJ: tight junction
tPa: tissue‐type plasminogen activator
TQ: transport quotient
ZO‐1: zonula occludens‐1

## 1 INTRODUCTION

The majority of strokes are caused by a clot of blood obstructing a blood vessel in the brain, leading to oxygen and glucose deprivation and finally neuronal death.^1^ Usually, a drug containing the endogenous serine protease tissue‐type plasminogen activator (tPa) is applied during early stages of ischemic stroke. Initially tPa was discovered as a part of the coagulation system and can be used therapeutically to dissolve blood clots resulting in a reperfusion of the affected brain.^2^ Despite its therapeutic effects, a severe side effect of tPa application is inducing different types of bleeding.^3^ Cranial hemorrhage is a serious complication of stroke and is related to blood‐brain barrier (BBB) breakdown. BBB disruption is detectable as early as 3 h after stroke onset and is accompanied by the degradation of tight junction (TJ) proteins.^4^, ^5^ The BBB limits paracellular diffusion of solutes by forming an impermeable barrier providing a strictly regulated microenvironment, essential for neuronal function.^6^ An important characteristic of the BBB is a high expression of TJ proteins like ZO‐1, Occludin and Claudin‐5 sealing the paracellular space between endothelial cells.^7^ Previously, it has been shown that the risk of cranial hemorrhage is increased after tPa application.^1^, ^8^ Although the mechanisms how tPa favors BBB disruption are still under debate, an interaction with the low‐density lipoprotein receptor‐related protein 1 (LRP1) has been proposed. LRP1 is a member of the low‐density lipoprotein receptor protein family and is involved in ligand endocytosis, transcytosis, and cell signaling. In the brain, LRP1 can be detected in neurons, microglia and within the BBB. In recent years many interactions of LRP1 with tPa have been identified.^9^ Circulating tPa can be transported via LRP1 across the BBB into the brain.^10^ Additionally, an upregulation of matrix metalloproteinase 9 (MMP9) after tPa/LRP1 interaction in the brain parenchyma has been observed under ischemic conditions.^11^ MMPs are inducible endopeptidases cleaving various components of extracellular matrix and BBB, including Claudin‐5.^12^ Although the main source of MMP9 remains unclear, a secretion from endothelial and activated microglial cells has been documented.^13^ These correlations might also be crucial for tPa‐ and LRP1‐related BBB degradation during stroke therapy. Based on these observations we hypothesized that the inhibition of tPa/LRP1 interaction might reduce tPa transport into the brain parenchyma and consequently tPa‐initiated side effects during acute stroke therapy.

## 2 MATERIAL AND METHODS

### 2.1 Antibodies

Rabbit anti‐β‐Actin (A2066, Sigma‐Aldrich, 1:1000), rabbit anti‐Claudin‐5 (34‐1600, Invitrogen, 1:1000), rabbit anti‐tPa (10147‐1‐AP, Proteintech, 1:2000), rabbit anti‐MMP9 (3852 S, Cell Signaling, 1:1000), rabbit anti‐ZO‐1 (sc‐10 804, Santa Cruz, 1:1000), mouse anti‐Occludin (33‐1500, Invitrogen, 1:1000), mouse anti‐LRP1 domains II/IV (11E2),^14^ rabbit anti‐Iba‐1 (17 198, Cell Signaling, 1:1000), nonspecific mouse IgG (31,903, ThermoFisher), HRP‐conjugated goat anti‐rabbit (A5278, Sigma‐Aldrich, 1:10 000), HRP‐conjugated donkey anti‐mouse (715‐035‐151, Jackson Immunoresearch, 1:10 000).

### 2.2 Antibody purification

Mouse anti‐LRP1 antibody (11E2) was purified from hybridoma cell supernatants using MINI Affi‐Prep® Protein A columns (Bio‐Rad) according to the manufacturer's protocol.

### 2.3 Radiolabeling of tPa

Radiolabeling of tPa was performed via indirect iodination using Na^125^I 1 mCi (Perkin‐Elmer) and Pierce™ IODOGEN tubes (Thermo scientific) according to the manufacturer's protocol.

### 2.4 Cell culture

For bEnd.3 and primary brain mouse endothelial cells (BMECs) DMEM, high glucose (Lonza) containing 10% (v/v) fetal bovine serum, 100 U/mL penicillin and 100 μg/mL streptomycin (all from Gibco) was used. α‐MEM (Lonza) supplemented with 10% (v/v) fetal bovine serum, 100 U/mL penicillin and 100 μg/mL streptomycin was used for CHO cell cultivation. Isolation of BMECs was performed as previously described^14^ using 8−9 weeks‐old wildtype mice (both sexes), which were housed on a 12 h:12 h‐light:dark cycle with ad libitium access to water and standard laboratory diet. Isolated cells were seeded on 24‐well ThinCert™ cell culture inserts (pore size, 0.4 μm; surface area, 33.6 mm^2^, Greiner Bio‐One), which were coated with 0.4 mg/mL collagen IV and 0.1 mg/mL fibronectin (both from Sigma‐Aldrich). Cultures were maintained in DMEM, high glucose supplemented with 20% (v/v) plasma‐derived bovine serum (First Link), 100 U/mL penicillin and 100 μg/mL streptomycin, 2 mmol/l l‐glutamine (Gibco), and 30 µg/mL endothelial cell growth supplement (Sigma‐Aldrich). During the first 2 days of in vitro culture, microvascular endothelial cells were selected using 4 μg/mL puromycin (Alexis). Cell growth was monitored using the cellZscope device (nanoAnalytics). When cells reached a capacitance < 1 μF/cm^2^, indicating monolayer formation, they were incubated with serum‐free culture media containing 550 nmol/l hydrocortisone (Sigma‐Aldrich) for 18 h to stimulate TJ formation.

Isolation of primary microglial cells was performed as described before^15^ sacrificing p1 wildtype mice (both sexes) housed on a 12 h:12 h‐light:dark cycle. All cells were cultured at 37°C and 5% CO_2_.

### 2.5 Protein extraction, SDS‐PAGE and immunoblotting

Cells were mechanically detached and lysed with cell lysis buffer (50 mmol/l Tris, 150 mmol/l NaCl, 0.02% [w/v] NaN_3_, 1% [v/v] Nonidet P‐40 supplemented with EDTA‐free protease inhibitor cocktail [cOmplete™, Roche Applied Science]). Protein concentrations of the lysates were measured using BCA assay according to the manufacturer's protocol (ThermoFisher). SDS‐Page and immunoblotting were performed with 20 µg (bEnd.3 and CHO) or 30 µg (microglia) lysate. Densitometric analyses of immunoblotting signals were performed using the ImageJ software (Version 1.52 q) followed by protein level normalization to β‐Actin signal intensities.

### 2.6 Concentrations

All experiments were conducted with 270 nmol/l tPa and 100 mg/mL of 11E2 antibody or nonspecific IgG.

### 2.7 Protein levels after tPa incubation

At confluency, bEnd.3 or microglial cells were incubated with culture media containing or lacking tPa (Alteplase, Boehringer) for 1 h. Afterwards, media was replaced with normal culture media. After 24 h cells were carefully washed with PBS pH 7.4, followed by PBS pH 2 treatment to remove membrane‐bound proteins. Proteins were extracted and analyzed as described before. The ionized calcium‐binding adapter molecule 1 (Iba‐1) was used as a marker for activated microglial cells.

### 2.8 MMP9 activity after tPa incubation

To assess MMP9 levels and activity after 1 h of tPa or negative control incubation as described before, conditioned media was collected 24 h after media change and directly analyzed via zymography using Novex 10% Zymogram Plus (Gelatin) gels (Invitrogen Life Technologies) according to manufacturer's instructions. Densitometric analyses of the individual bands were performed as described above. Band intensities were normalized to protein amounts loaded onto the gel, which were revealed by BCA assay as described above.

### 2.9 In vitro transcytosis studies

Barrier properties of endothelial cells were estimated by measurement of the transendothelial electrical resistance (TEER). bEnd.3 cells were seeded on coated cell culture inserts and TJ protein formation was stimulated as described before. TEER values were monitored using the cellZscope device. Stimulated cells were incubated with or without tPa. After 1 h media was replaced with serum‐free culture media and TEER values were tracked for 24 h. Moreover, the serum‐free culture media of the luminal compartment was supplemented with 50 µg/mL fluorescein isothiocyanate (FITC)‐Dextran (molecular weight 3000−4000, Sigma‐Aldrich). To assess paracellular leakage across the endothelial monolayer, fluorescence intensities of FITC‐dextran in the abluminal and luminal compartments were measured 24 h later using the Varioskan LUX multimode microplate reader (SkanIt Software 6.0.2.3).

### 2.10 LRP1‐mediated tPa internalization

Internalization of tPa was analyzed by comparing CHO K1 cells with the LRP1‐deficient CHO 13‐5‐1 cell line. At confluency, cells were incubated with serum‐free culture media containing tPa and LRP1‐blocking antibody 11E2 or nonspecific IgG for 1 h. Protein extraction, SDS‐PAGE, and densitometric analyses were performed as described before.

### 2.11 LRP1‐mediated tPa transport

To analyze tPa transports, isolated BMEC cells were plated on coated cell culture inserts as described before. Radiolabeled [^125^I]–tPa, 11E2 antibody and 1 µCi/mL of the paracellular diffusion marker [^14^C]–inulin (Perkin‐Elmer) were added to the luminal compartment. After 1 h, luminal and abluminal media was collected and TCA precipitation was performed with 20% (v/v) TCA followed by centrifugation at 4°C for 20 min at 20.000 RCF. Protein‐bound [^125^I] was analyzed using the Wizard2 Gamma Counter (Perkin Elmer), whereas [^14^C]‐inulin was measured with the Tri‐Carb 2800 TR Liquid Scintillation Analyzer (Perkin‐Elmer). The transport quotient (TQ) of [^125^I] – tPa was calculated as follows:

tPaTQ=([I125]–tPaabluminal/[I125]–tPainput)/([C14]−inulinabluminal/[C14]−inulininput)

### 2.12 Primary microglia/bEnd.3 semi coculture model

To analyze effects of transported tPa on the activation status of microglial cells, protein levels and activity of MMP9, a semi coculture model was established. Primary microglia were isolated as described before. bEnd.3 cells were seeded on coated cell culture inserts and TEER was monitored. At the time of TJ stimulation, microglial cells were seeded, bEnd.3 cells were divided into three groups similar in TEER and capacitance values and cultured in serum‐free culture media with or without tPa and with or without 11E2 for 1 h. Subsequently, abluminal conditioned media was transferred to the primary microglial cells. After 1 h, media was replaced with regular culture medium. 24 h later microglial cells were lysed as described before and supernatants collected for zymography. SDS‐Page, immunoblotting, zymography and densitometric analyses were performed as defined before. In another set of experiments, microglial cells were seeded on 24‐well plates. Culture inserts with formed and stimulated bEnd.3 cell monolayer as described before were transferred to the wells of the 24‐well plates, resulting in a defined luminal compartment and abluminal compartment containing the primary microglial cells. The luminal serum‐free culture media was supplemented with or without tPa and with or without 11E2. Luminal media was replaced 1 h later with regular culture media containing 75 µg/mL FITC‐Dextran (molecular weight 3000−4000). After 24 h, either culture inserts were transferred to the cellZscope device for TEER monitoring or luminal or abluminal media were collected for FITC‐Dextran fluorescence analyses as described before.

### 2.13 Statistical analysis

For statistical analysis GraphPad Prism (8.4.3) was used. Final images were arranged using CorelDRAW2018. Schematic illustrations were created with bioRender.com.

## 3 RESULTS

**Figure 1:**
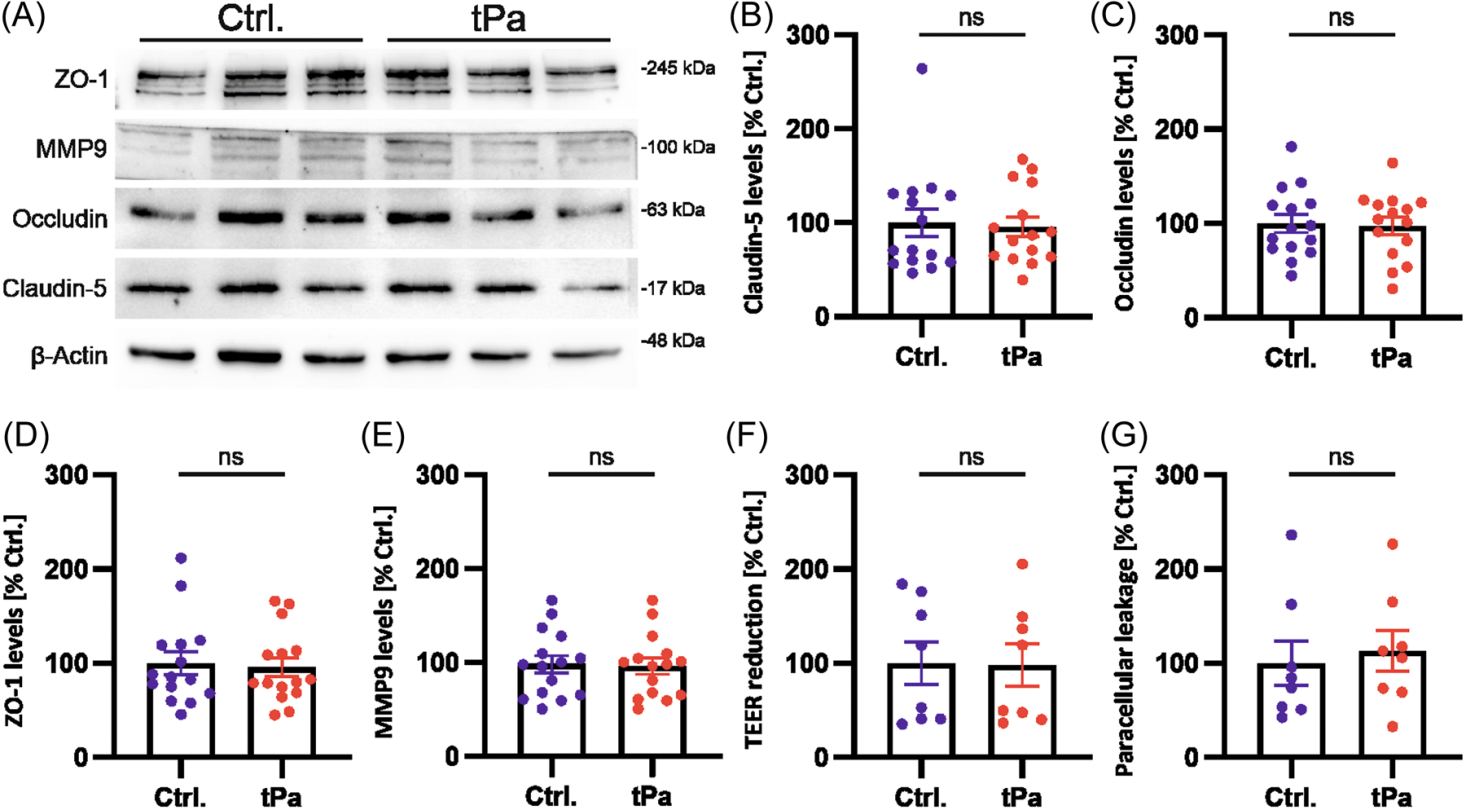
bEnd.3 barrier properties did not change after tPa incubation. (A) Representative immunoblotting for protein levels in cell lysates of bEnd.3 cells 24 h after incubation with tPa for 1 h compared to untreated cells. (B−E) Claudin‐5, Occludin, ZO‐1 and MMP9 protein levels were quantified by densitometric analysis after immunoblotting and normalized to ß‐actin. (F) TEER values were measured before tPa incubation and 24 h afterwards. Percental reduction of TEER values over 24 h was calculated. (G) Paracellular leakage levels of FITC‐Dextran 24 h after tPa incubation of bEnd.3 cells for 1 h. All data were normalized to the untreated control cells. Data represent individual values and mean ± SEM of *n* = 15 (B−E) or 8 (F + G) individual replicates for both treated and untreated group from five (B−E) or one (F + G) experiment. Unpaired *t*‐test was used for statistical analysis (*p < 0.05). FITC, fluorescein isothiocyanate; MMP9, matrix metalloproteinase 9; SEM, standard error of the mean; TEER, transendothelial electrical resistance.

In an in vitro proof of concept study we analyzed whether the cellular levels of MMP9 or the TJ proteins ZO‐1, Occludin and Claudin‐5 are affected by tPa. Therefore, we applied therapeutic concentrations of tPa to bEnd.3 cells for 1 h and analyzed the respective protein amount 24 h later. We did not detect tPa‐related changes in Claudin‐5 (*p* = 0.9), Occludin (*p* = 0.9), ZO‐1 (*p* = 0.8) and MMP9 levels (*p* = 0.9) (Figure 1B−E). To investigate effects on barrier properties, we monitored TEER, an in vitro parameter reflecting the integrity of endothelial monolayers, using the same experimental setup. No significant difference in TEER values (*p* = 0.9) was detectable comparing the tPa‐treated and untreated control group 24 h after tPa treatment (Figure 1F). Additionally, paracellular leakage was assessed using FITC‐Dextran, a carbohydrate with no known channel or receptor. Paracellular leakage of FITC‐Dextran averaged out at 0.3% 24 h after application, irrespective of tPa treatment (*p* = 0.7) (Figure 1G).

**Figure 2:**
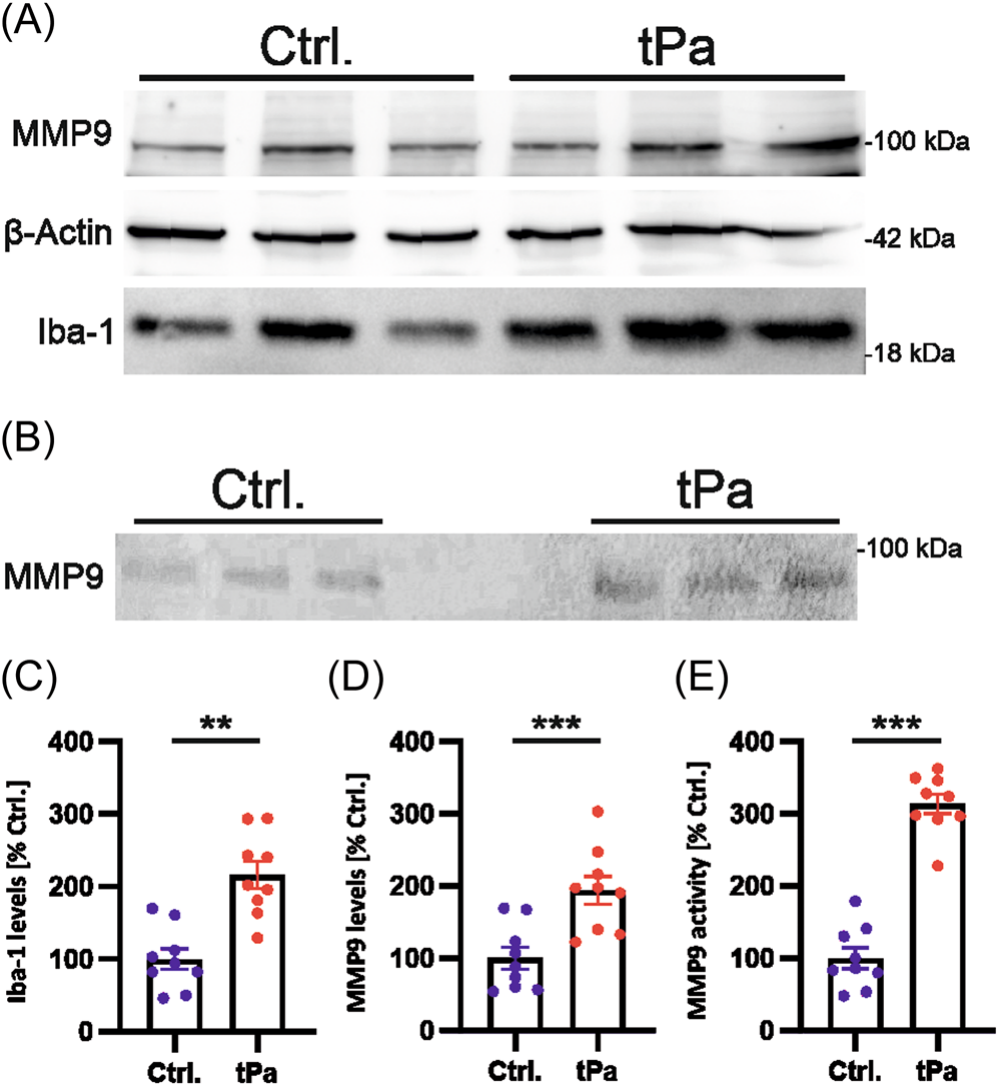
Microglial activation, elevated MMP9 levels and activity after tPa incubation. (A) Representative immunoblotting for MMP9 and Iba‐1 levels in cell lysates of primary microglial cells 24 h after incubation with tPa for 1 h compared to untreated cells. (B) Representative zymography gelatin gel for MMP9 activity in microglial supernatants 24 h after incubation with tPa for 1 h compared to untreated cells. (C) Iba‐1 and (D) MMP9 levels were quantified by densitometric analysis after immunoblotting and normalized to ß‐actin. (E) MMP9 activity was quantified by densitometric analysis of zymography gels and normalized to loaded protein amount. Signal intensities of untreated control cells were set to 100%. Data represent individual values and mean ± SEM of *n* = 9 individual replicates for both treated and untreated group from three independent experiments. Unpaired *t*‐test was used for statistical analysis (*p < 0.05; **p < 0.01; ***p < 0.001). SEM, standard error of the mean.

According to previous studies, we investigated tPa sensitivity of primary microglial cells regarding activation and alterations in MMP9 protein levels and activity (Figure 2A,B). tPa incubation at therapeutic concentration led to a significant increase in Iba‐1 levels (*p* = 0.0002) compared to untreated cells, indicating microglial activation (Figure 2C). Accompanying, significantly elevated cellular MMP9 levels (*p* = 0.0015) and MMP9 activity in microglial supernatants (*p* < 0.0001) were detected when compared to untreated cells (Figure 2D,E).

**Figure 3:**
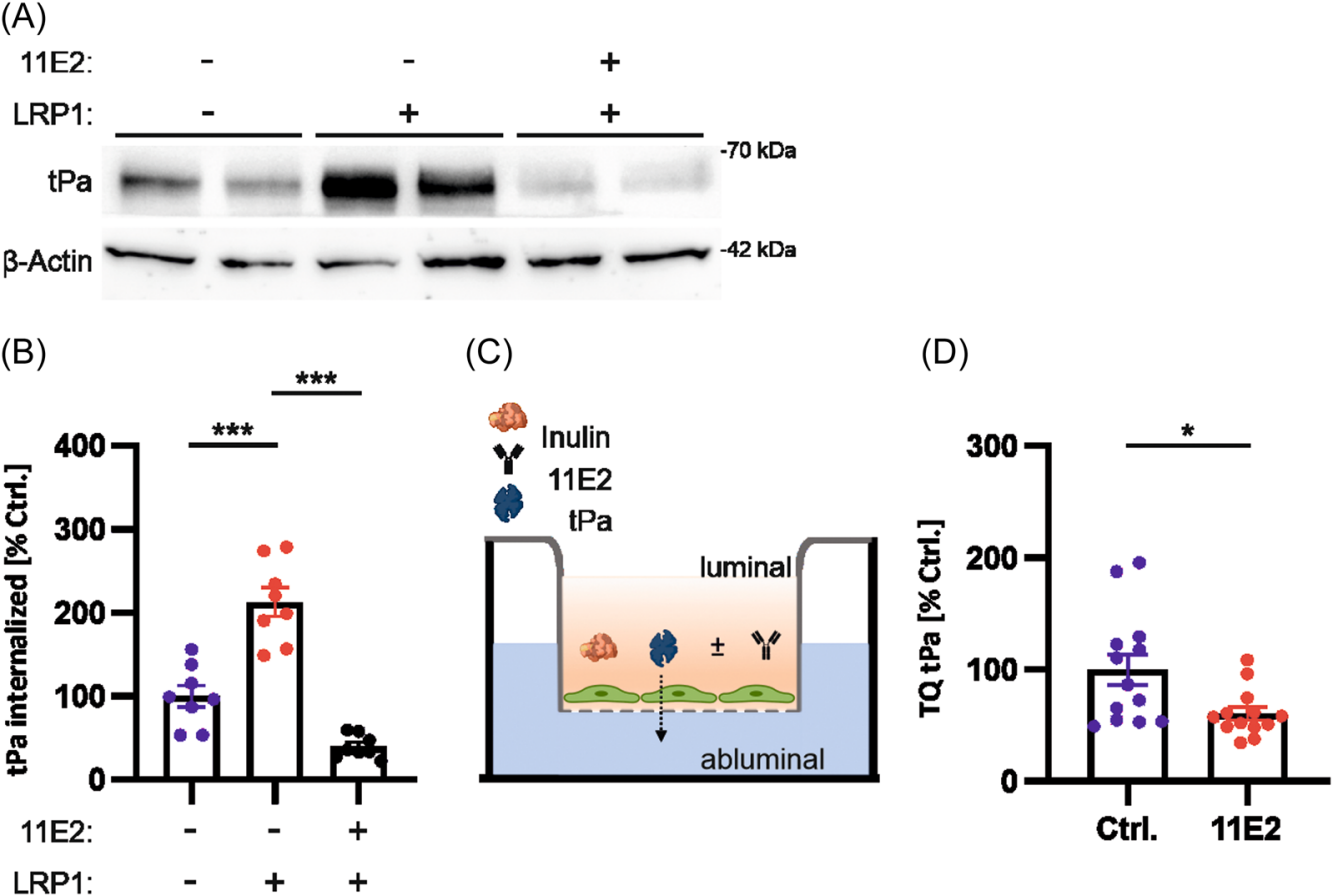
Reduction of tPa internalization and transport in presence of anti‐LRP1 antibody 11E2. (A) Representative immunoblotting for tPa in cell lysates of CHO K1 and CHO 13‐5‐1 cells after incubation with tPa for 1 h. LRP1‐deficient CHO 13‐5‐1 cells were used as negative control. LRP1‐expressing CHO K1 cells incubated with 11E2 antibody were compared to cells incubated with nonspecific IgG. (B) tPa levels were quantified by densitometric analysis after immunoblotting and normalized to ß‐actin. Untreated LRP1‐deficient cells were set to 100%. (C) Schematic illustration of [^125^I]–tPa transport across a monolayer (D) Transport of [^125^I]–tPa with or without 11E2 was performed in BMECs for 1 h. TQ from luminal to abluminal compartment was calculated by relating the transported [^125^I]–tPa to diffused [^14^C]–inulin. TQ was normalized to untreated control cells. Data represent individual values and mean ± SEM of n = 8 (B) or 13 (D) individual replicates for both treated and untreated group from four (B) or one (D) experiment. One‐way ANOVA followed by Tukey's multiple comparison test (B) or unpaired *t*‐test (D) was used for statistical analysis (*p < 0.05; **p < 0.01; ***p < 0.001). ANOVA, analysis of variance; SEM, standard error of the mean.

Since LRP1 was identified as the main transport receptor for tPa within the BBB, we assumed that blocking tPa binding to LRP1 may reduce tPa translocation from the blood into the brain. Previously, we developed the anti‐LRP1 antibody 11E2, sharing its LRP1 binding sites with the most known LRP1 ligands.^14^ Accordingly, we could show, that tPa internalization was significantly reduced by ~81% in cells highly expressing LRP1 (CHO K1) (*p* < 0.0001) in the presence of 11E2 compared to nonspecific IgG (Figure 3A,B). The antibody's ability to reduce tPa transport across an endothelial barrier was verified using primary BMECs (Figure 3C). Thereby, tPa transport across BMEC monolayers was significantly reduced by ~39% (*p* = 0.01) in the presence of 11E2 antibody compared to nontreated cells (Figure 3D).

**Figure 4:**
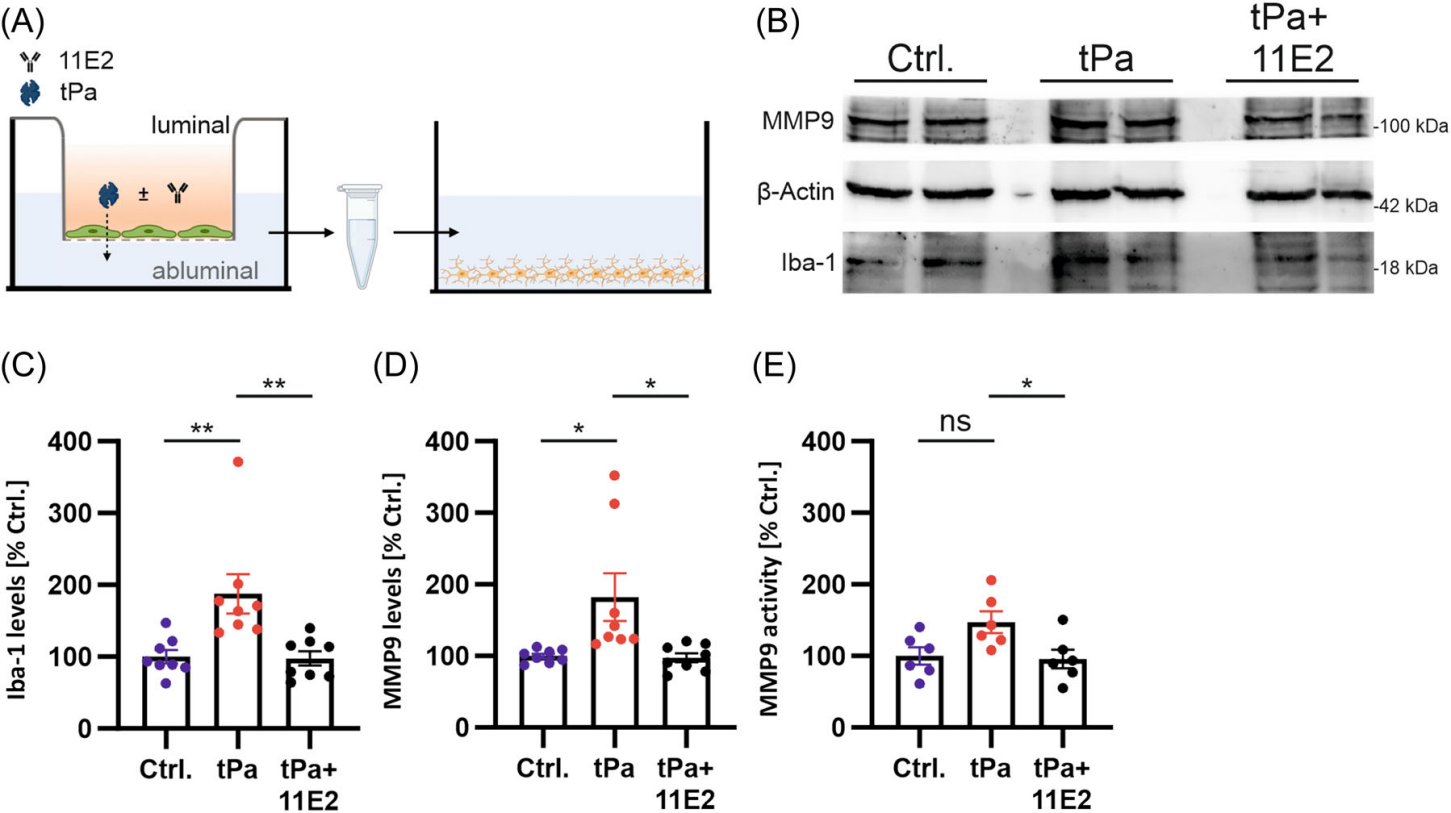
tPa induced elevated MMP9 and Iba‐1 levels in microglia after LRP1‐mediated transcytosis across bEnd.3 cells. (A) Schematic illustration of the semi coculture model. (B) Representative immunoblotting for protein levels in cell lysates of primary microglial cells 24 h after incubation with abluminal supernatants of bEnd.3 cells incubated with tPa with or without 11E2 for 1 h compared to untreated cells. (C) Iba‐1 and (D) MMP9 levels were quantified by densitometric analysis after immunoblotting and normalized to ß‐actin. (E) MMP9 levels of microglial supernatants were quantified by densitometric analysis of zymography gels and normalized to the protein amount loaded onto the gel. Intensities of untreated control cells were set to 100%. Data represent individual values and mean ± SEM of *n* = 8 (C + D) or 6 (E) individual replicates for both treated and untreated group from four (C + D) or three (E) independent experiments. One‐way ANOVA followed by Tukey's multiple comparison test was used for statistical analysis (*p < 0.05; **p < 0.01). ANOVA, analysis of variance; SEM, standard error of the mean.

Based on our previous results, we analyzed the impact of the LRP1‐blocking 11E2 antibody coapplication during tPa treatment on primary microglial cells in a semi coculture model. We performed tPa transport across an endothelial barrier and after 1 h, microglial cells were incubated with conditioned medium from the abluminal compartment of the endothelial monolayer. Respective protein amount was analyzed 24 h later (Figure 4A). Application of therapeutic concentrations of tPa on bEnd.3 cells induced a significant increase in cellular Iba‐1 (*p* = 0.0056/*p* = 0.0045) and MMP9 (*p* = 0.0196/*p* = 0.016) levels in microglial cells compared to untreated cells and to a coapplication with 11E2 antibody. However, no such changes were apparent in the presence of 11E2 (*p* = 0.995/*p* = 0.995) compared to untreated control group (Figure 4B−D). Similar to the increased protein level, MMP9 activity in microglial supernatants was significantly increased when bEnd.3 cells were incubated with therapeutic concentration of tPa compared to cells incubated with tPa and 11E2 antibody (*p* = 0.04). Incubation of endothelial cells with tPa or tPa and 11E2 antibody did not change MMP9 activity compared to untreated cells (*p* = 0.0619/*p* = 0.972) (Figure 4E).

**Figure 5:**
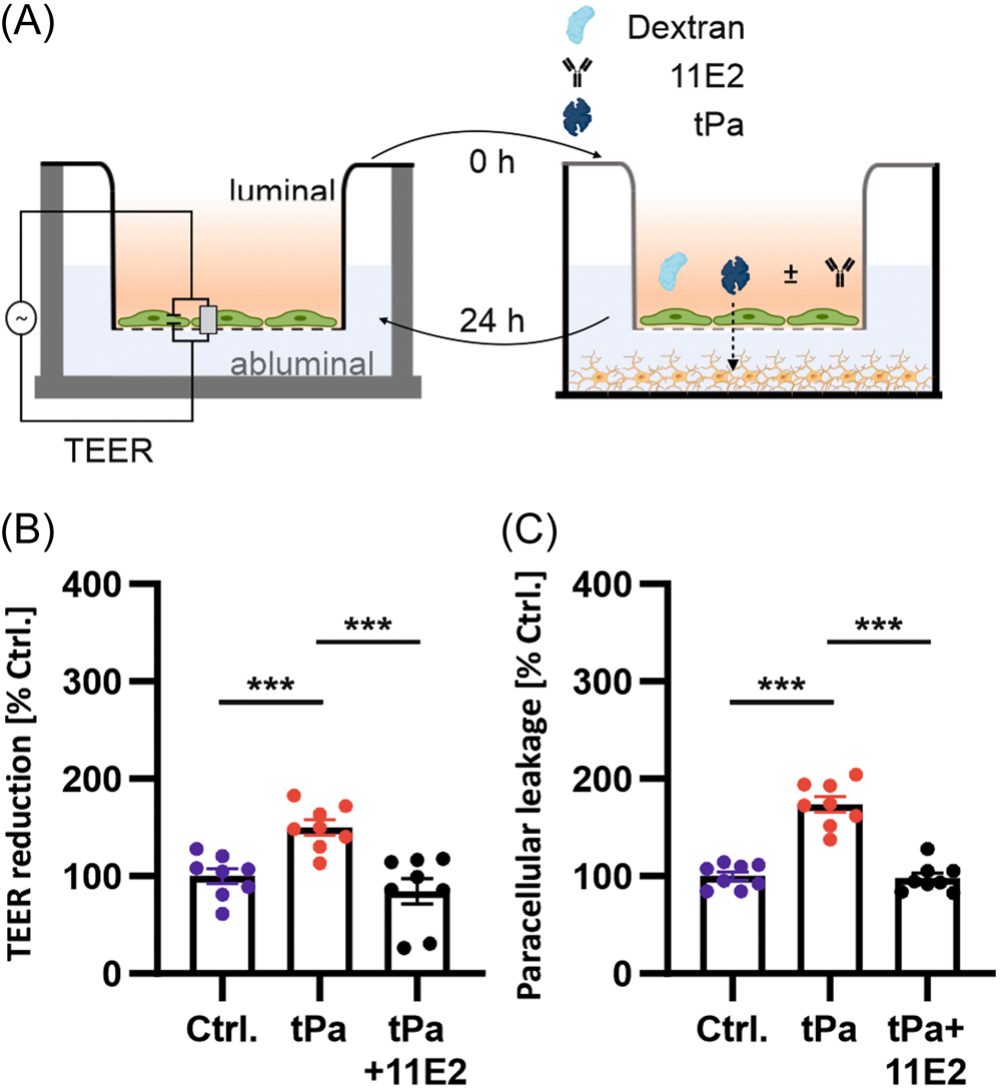
tPa induced changes in barrier properties after LRP1‐mediated transcytosis across bEnd.3 cells. (A) Schematic illustration of the coculture model (B) TEER values were measured before tPa incubation and 24 h afterwards, percental reduction of TEER values over 24 h was calculated. (C) Paracellular leakage of FITC‐Dextran 24 h after tPa incubation of bEnd.3 cells for 1 h. Values (B) and averaged paracellular leakage level (C) of the untreated control group was set to 100%. Data (B + C) represent individual values and mean ± SEM of *n* = 8 individual replicates for both treated and untreated group from two independent experiments. One‐way ANOVA followed by Tukey's multiple comparison test was used for statistical analysis (***p < 0.001). ANOVA, analysis of variance; FITC, fluorescein isothiocyanate; SEM, standard error of the mean; TEER, transendothelial electrical resistance.

To validate our hypothesis that tPa contributes to BBB breakdown after LRP1‐dependent transcytosis, we analyzed the 11E2 antibody's capability to reduce tPa‐mediated barrier disruption in a direct coculture model as described above (Figure 5A). Cocultures of bEnd.3 and microglial cells treated for 24 h with therapeutic concentrated tPa showed significantly reduced TEER values and paracellular diffusion compared to untreated cocultures (*p* = 0.0047/*p* < 0.0001) or cocultures additionally incubated with 11E2 antibody (*p* = 0.0003/*p* < 0.0001). The coapplication of LRP1‐blocking antibody 11E2 preserved barrier functionality in the presence of tPa regarding TEER and paracellular leakage levels compared to untreated cocultures (*p* = 0.519/*p* = 0.975) (Figure 5B,C).

## 4 DISCUSSION

An important mechanism in the pathogenesis of ischemic stroke is BBB breakdown, which is a precursor for stroke‐related complications like hemorrhagic transformation.^2^, ^16^ Loss of BBB integrity is accompanied by the degradation of TJ proteins, predominantly of Claudin‐5.^5^ Although many factors contribute to an increased TJ protein degradation, MMPs seem to play a pivotal role especially at the earliest stage of stroke onset.^17^ However, in the presence of tPa, an accelerated BBB breakdown can be observed.^16^ This effect is based presumably on the interaction of tPa with LRP1.^3^, ^18^ In fact, there is evidence that the application of tPa during stroke therapy increases MMP9 levels in a LRP1‐dependent fashion.^9^, ^19^ Consequently, increased MMP9 activity within ischemic tissue correlates with the degradation of Claudin‐5, an essential component of barrier functionality. However, the source of extracellular MMP9 remains controversial. In the present in vitro study we did not observe any changes neither in MMP9, nor in TJ protein levels analyzing the endothelial cell line bEnd.3 after incubation with high therapeutic concentrations of tPa (Figure 1A−E). Additionally, the cell monolayer displayed no tPa‐dependent changes in barrier properties regarding TEER values and paracellular diffusion (Figure 1F,G). Since we did not observe any direct effects of tPa treatment on endothelial cells we hypothesized that an interaction with cells located within the separated brain parenchyma (e.g., microglia) is necessary to initiate barrier disruption. In fact, we were able to detect elevated activation of microglial cells, increased cellular MMP9 levels as well as an increase in circulating MMP9 activity due to direct tPa treatment (Figure 2C−E). Our results indicate that the translocation of therapeutically used tPa from the blood into the brain might lead to an activation of microglial cells, resulting in an increased activity of MMP9. Since tPa is transported across the BBB LRP1‐dependently, a limitation of tPa transport by coapplication of a LRP1‐blocking antibody might diminish BBB breakdown by tPa‐induced microglia activation. In the present study we were able to reduce tPa uptake and transport in BMECs by the application of the anti‐LRP1 antibody 11E2 (Figure 3B,D). With our indirect and direct coculture models we provide evidence that tPa transported across an endothelial monolayer enhances microglial activation as well as MMP9 levels (Figure 4C−E). tPa‐mediated microglial cell activation is followed by endothelial monolayer breakdown, which was verified by reduced TEER as well as increased paracellular leakage levels (Figure 5B,C). Since we demonstrated that the inhibition of tPa/LRP1 interaction at the luminal surface prevented microglial activation and consequently preserved BBB integrity, our findings show for the first time the therapeutical benefit of the specific LRP1 inhibition by monoclonal antibodies (Figure 5B,C). In similar studies, tPa/LPR1 interaction was blocked by the coapplication of the receptor associated protein (RAP), a chaperon binding to all members of the low‐density lipoprotein receptor protein family.^20^ Consistently, the inhibition of tPa/LRP1 interaction prevented tPa transport across the BBB, decreased activation of microglia and subsequent MMP9 release and cytokines and finally attenuated disruption of BBB responsible for hemorrhagic transformation following stroke.^9^ Although RAP is a potent LRP1 inhibitor preserving tPa‐related BBB breakdown, due to its broad range of interaction partners, RAP seems poorly suited for the therapeutic application in humans. Importantly, RAP is required for the proper folding and maturation of several receptors of the LDL receptor family such as (very) low density lipoprotein receptor (LDLR), LRPs and apolipoprotein E receptor 2, but also acts as an antagonist for several interaction partners such as blood coagulation factors, insulin or apolipoprotein E. Consequently, the usage of RAP as therapeutic agent might affect fundamental cellular signaling pathways involved in lipoprotein metabolism, energy homeostasis, organogenesis, blood coagulation and neuronal regeneration, which might cause significant side effects. In this context, impaired LDLR function is associated with hypercholesterolemia and coronary artery diseases, while loss of function of LRP5 is involved in osteoporosis pseudoglioma and impaired insulin secretion.^21^ In contrast, the application of monoclonal antibodies sequestering exclusively the specific target is an established form of treatment verified in terms of efficiency and safety for many years. In line with this, we provide a potential selective therapeutic option via the monoclonal anti‐LRP1 antibody 11E2 that inhibits specifically the tPa/LRP1 interaction without interfering with other receptors. Since the binding of tPa to LRP1 is independent from tPa's catalytic region, the specific blockage of the binding site could not only reduce severe tPa‐associated side effects but also maintain the catalytic function of tPa, essential for dissolving blood clots. Taking into consideration that the major degradation pathway of peripheral tPa is the constant LRP1‐mediated endocytosis via the liver, the coapplication of LRP1‐blocking antibodies might prolong the presence of high therapeutic tPa concentrations within the vascular system and its effects. However, since LRP1 is a ubiquitously expressed cell surface receptor, the coapplication of LRP1‐blocking antibodies during acute stroke therapy could still be accompanied by side effects. Further in vivo studies will be necessary to proof the in vitro observations and clarify whether the inhibition of tPa‐mediated BBB breakdown outweighs possible systemic side effects.

## AUTHOR CONTRIBUTIONS

Philip H. Klecker (ORCiD: 0000‐0002‐6735‐2970) and Laura Fritzen (ORCiD: 0000‐0002‐2094‐6368) designed the studies, conducted all experiments, wrote the manuscript and contributed equally to this work. Alexander D. Mazura (ORCiD: 0000‐0002‐2899‐6183) and Claus U. Pietrzik supervised the experimental design and entire work of the manuscript. All authors read and approved the final manuscript.

## CONFLICT OF INTEREST STATEMENT

The authors declare no conflict of interest.

## ETHICS STATEMENT

All animal studies were conducted in compliance with European and German guidelines for the care and use of laboratory animals and were approved by the Central Animal Facility of the University of Mainz and the ethical committee on animal care and use of Rhineland‐Palatinate, Germany.

## Supporting information

Supporting information.

Supporting information.

## Data Availability

All data generated or analyzed during this study are included in this published article and its supplementary information files.
