# Supplementary material for: Antibody‐mediated inhibition of tissue‐type plasminogen activator binding to the low‐density lipoprotein receptor‐related protein 1 as a potential beneficial modulator for stroke therapy
Source: J Cell Biochem. 2023 Jun 8;124(7):1040–9. doi: 10.1002/jcb.30431 (PMC13484565; doi:10.1002/jcb.30431)
Supplement: Supplementary file 1 [file JCB-124--s002.pdf]

Application: Tecan i-control	Tecan i-control , 2.0.10.0	
Device: infinite 200Pro	Serial number: 1801008219	Serial number of connected stacker:

Firmware: V_5.26_10/17_InfiniteRX (Oct 19 201 MAI, V_5.26_10/17_InfiniteRX (Oct 19 2017/13.54.43)

Date: 25.05.22
Time: 12:15:42 PM

System	DESKTOP-9BTE17V
User	DESKTOP-9BTE17V\LI-COR_IMAN
Plate	Greiner 96 Flat Bottom Transparent Polystyrene Cat. No.: 655101/655161/655192 [GRE96ft.pdfx]
Plate-ID (Stacker)	

Label: BCA

Mode	Absorbance	Standard 1 BLANK
Measurement Wavelength	562 nm	Standard 2 1 µg/µl BSA
Bandwidth	9 nm	Standard 3 2 µg/µl BSA
Number of Flashes	25	Standard 4 3 µg/µl BSA
Settle Time	0 ms	Standard 5 4 µg/µl BSA
Part of Plate	A1-B12	Standard 6 5 µg/µl BSA
Start Time: 5/25/2022 12:15:42 PM		
End Time: 5/25/2022 12:16:05 PM		
Movement	Move Plate Out	

Optical density (OD)

Temperature: 19 °C

<>	Standard 1	Standard 2	Standard 3	Standard 4	Standard 5	Standard 6	Sample 1	Sample 2	Sample 3	Sample 4	Sample 5	Sample 6	
A	0,1	0,3194	0,4774	0,64	0,7861	0,9148	0,4767	0,2929	0,3181	0,4296	0,4476	0,3301	
B	0,106	0,3372	0,5066	0,6833	0,8362	0,9627	0,5289	0,3256	0,3504	0,4747	0,4845	0,3549	
	0,103	0,3283	0,492	0,66165	0,81115	0,93875	0,5028	0,30925	0,33425	0,45215	0,46605	0,3425	MEAN
		0,2253	0,389	0,55865	0,70815	0,83575	0,3998	0,20625	0,23125	0,34915	0,36305	0,2395	Substraction BLANK

Standard curve

BSA Standar	OD
1	0,2253
2	0,389
3	0,55865
4	0,70815
5	0,83575

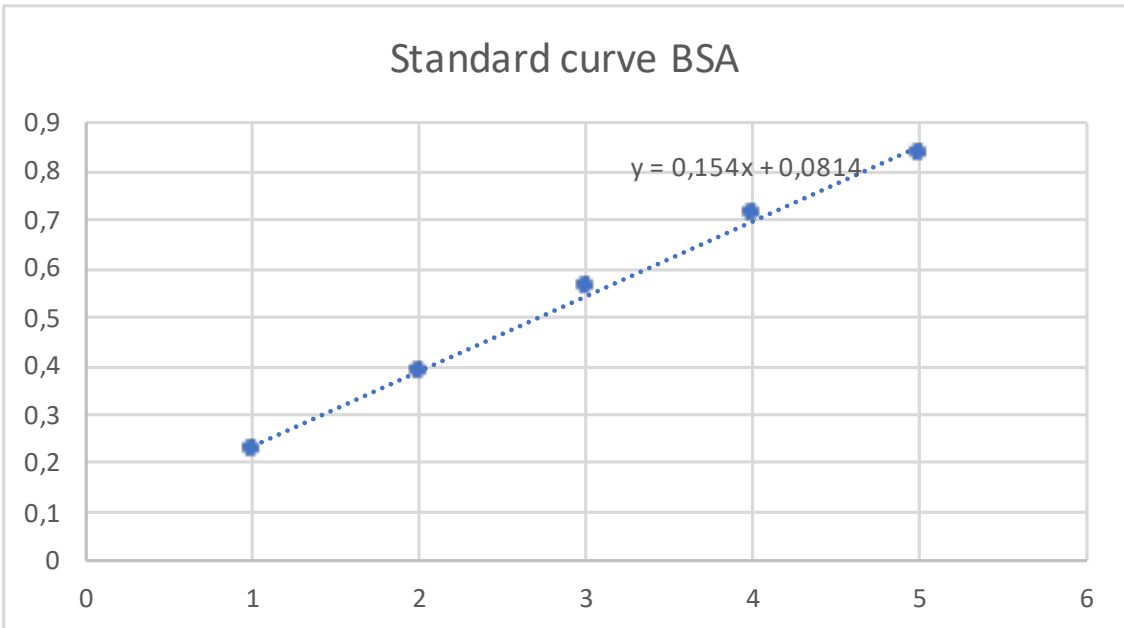

Formular: y = 0,154x + 0,0814
x = (y - 0,0814)/0,154

Sample 1	Sample 2	Sample 3	Sample 4	Sample 5	Sample 6	
0,3998	0,20625	0,23125	0,34915	0,36305	0,2395	
0,3184	0,12485	0,14985	0,26775	0,28165	0,1581	Subsraction 0,0814
2,07	0,81	0,97	1,74	1,83	1,03	Division 0,154

protein concentration in µg/µl	
Sample 1	2,07
Sample 2	0,81
Sample 3	0,97
Sample 4	1,74
Sample 5	1,83
Sample 6	1,03
