# Supplementary material for: Antibody‐mediated inhibition of tissue‐type plasminogen activator binding to the low‐density lipoprotein receptor‐related protein 1 as a potential beneficial modulator for stroke therapy
Source: J Cell Biochem. 2023 Jun 8;124(7):1040–9. doi: 10.1002/jcb.30431 (PMC13484565; doi:10.1002/jcb.30431)
Supplement: Supplementary file 2 [file JCB-124--s001.docx]

**Information on quantification of immunoblotting results:**

Due to word limitation, further information on quantification will be provided in supplementary data files and not in the manuscript.

Protein concentrations of cell lysates and cell culture supernatants were determined using Piecre^TM^ BCA protein assay according to the manufacturer’s protocol (#23225, ThermoFisher). The optical density was measured at a wavelength of 562 nm using Tecan i-control infinite 200Pro (Version 2.0.10.0). Mean optical density from two duplicates was considered as final result. A standard curve of bovine serum albumin (BSA) ranging from 0 µg/µl to 5 µg/µl was generated. Based on the optical density of samples at a wavelength of 562 nm, protein concentrations (µg/µl) of cell lysates or cell culture supernatants were calculated using the formular of the generated linear standard curve. For this reason, we assumed a linear input-output relation regarding linearity check. Additionally, we provide a supplementary data file #2 containing an example of a calculation of protein concentrations using Piecre^TM^ BCA protein assay.

Membranes were incubated with secondary antibodies for one hour.

HRP-conjugated goat anti-rabbit (A5278, Sigma-Aldrich, Dilution 1:10,000),

HRP-conjugated donkey anti-mouse (715-035-151, Jackson Immunoresearch, Dilution 1:10,000)

For signal detection chemiluminescence assay was performed. For this purpose, the Immobilon®Western kit was used. The HRP substrate peroxide solution and the HRP substrate luminol reagent, were mixed in a 1:1 ratio and spread evenly on the membrane. The light signal was captured by the camera of the charge-coupled device (CCD) imager ChemiDoc MP (Version 3.0.1.14).

Quantification: Band intensities on nitrocellulose membranes or zymography gels were quantified using Image J software (Version 1.52 q). Thereby, rectangles were drawn around each band and a plot profile of each lane was made. The profile plot represents the relative density of the contents of the rectangle over each lane. The straight line tool was used to close off the base of each peak of interest. Afterwards, the area under each peak was calculated. Band intensities of bands corresponding to TJ proteins, tPa, Iba-1 and MMP9 were normalized to the respective signal strength of actin and are shown relative to control. Band intensities of MMP9 on zymography gels were normalized to protein amount loaded onto the gel and are shown relative to control.
